# Effect of Nanoparticle Incorporation and Surface Coating on Mechanical Properties of Bone Scaffolds: A Brief Review

**DOI:** 10.3390/jfb7030018

**Published:** 2016-07-12

**Authors:** Jesus Corona-Gomez, Xiongbiao Chen, Qiaoqin Yang

**Affiliations:** 1 Department of Mechanical Engineering, University of Saskatchewan, 57 Campus Drive, Saskatoon, SK S7H 5A9, Canada; jec489@mail.usask.ca (J.C.G.); xbc719@mail.usask.ca (X.C.); 2 Division of Biomedical Engineering, University of Saskatchewan, 57 Campus Drive, Saskatoon, SK S7H 5A9, Canada

**Keywords:** tissue engineering, mechanical properties, scaffold design, scaffold fabrication, biomaterials, nanodiamond, hydroxyapatite, bioactive glass particles, silver nanoparticles, surface coating

## Abstract

Mechanical properties of a scaffold play an important role in its in vivo performance in bone tissue engineering, due to the fact that implanted scaffolds are typically subjected to stress including compression, tension, torsion, and shearing. Unfortunately, not all the materials used to fabricate scaffolds are strong enough to mimic native bones. Extensive research has been conducted in order to increase scaffold strength and mechanical performance by incorporating nanoparticles and/or coatings. An incredible improvement has been achieved; and some outstanding examples are the usage of nanodiamond, hydroxyapatite, bioactive glass particles, SiO_2_, MgO, and silver nanoparticles. This review paper aims to present the results, to summarize significant findings, and to give perspective for future work, which could be beneficial to future bone tissue engineering.

## 1. Introduction

Mechanical properties of a scaffold play an important role on its in vivo performance after implantation, due to the fact that implanted scaffolds are typically subjected to different mechanical stresses including compression, tension, torsion, and shearing [1]. It is expected that the mechanical properties of a scaffold match with the ones of the native tissue to be repaired [1,2]. Toward this end, it is imperative to characterize the mechanical properties of scaffolds after their fabrication and before their implantation to ensure the appropriate performance [2,3,4,5,6,7]; otherwise, the implanted scaffold may not be succeeding in the following repair process.

Mechanical properties of scaffolds are of great importance in many tissue engineering applications and among them, the mechanical properties of scaffolds for bone tissue engineering, may be the most critical one. The important mechanical properties of bone include the toughness, Young’s modulus, tensile strength, compressive strength, shear modulus, and fatigue strength. Mimicking these characteristic mechanical properties combined with bone’s architecture at the macroscopic level are essential for a bone scaffold at the stage of implantation and afterwards, to maintain them appropriate for regeneration of new tissue. Many studies have been pursued with an aim at improving the mechanical properties of scaffolds by means of nanoparticles, surface coating, or the combination of both. A challenge involved in these studies is that the nanoparticles and/or coated materials used should be compatible with the tissue surrounded the implant, be able to promote cell adhesion, and be degraded gradually without toxicity to the patient.

This paper presents a brief review on the scaffold mechanical properties and their improvement, for bone tissue engineering, by means of nanoparticles and/or surface coating. Particularly, this paper focuses on the materials used for this purpose, including nanodiamond, hydroxyapatite, and bioactive glass, as well as the mechanical testing methods and the effects of nanoparticles and surface coating on the improved mechanical properties of scaffolds.

## 2. Nanoparticles and Coating Materials for Mechanical-Property Improvement

### 2.1. Nanodiamond

Nanodiamonds (NDs) represent a new class of nanoparticles in the carbon family, with excellent physical and chemical properties. NDs have valuable properties which make them a suitable option for increasing mechanical properties of scaffolds in tissue engineering applications [8]. Those properties include their nanoscale size, nearly spherical shape, rich surface chemistry, excellent biocompatibility, physico-chemical properties and low cytotoxicity [8,9,10,11]. The interactions of NDs with different kinds of cell cultures (and some tissues) have been studied for both pure NDs and NDs with biomolecule conjugates [12]. When adequately dispersed, NDs can increase the strength, toughness, and thermal stability of the nanocomposites [9].

**Figure 1:**
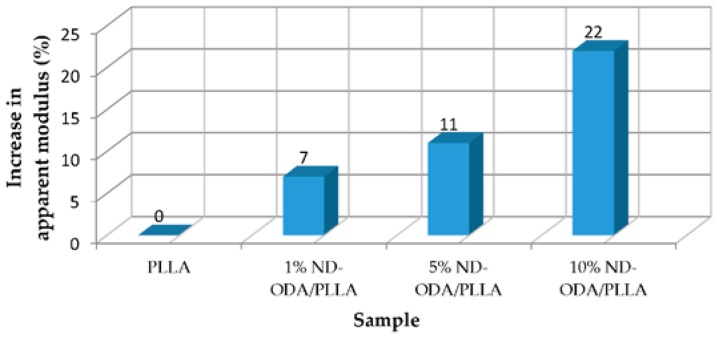
Improvement of apparent modulus in compression tests [8].

**Figure 2:**
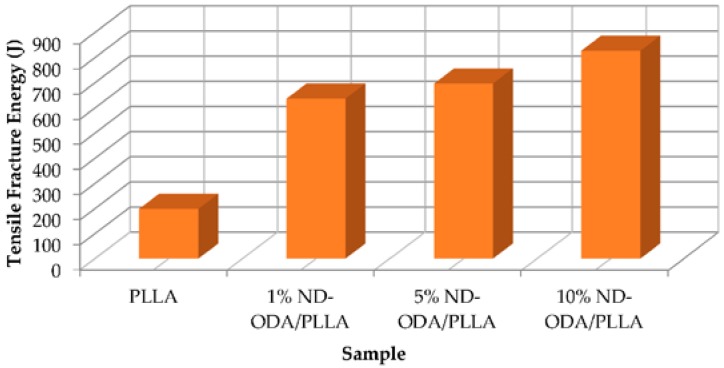
Fracture energy in tensile tests [8].

Zhang et al. [8] increased the mechanical properties of poly(l-lactic acid) (PLLA) scaffold by addition of octadecylamine-functionalized nanodiamond (ND-ODA). It was observed that with 10% wt of ND-ODA the strain at failure increased 280% and the fracture energy in tensile tests increased 310%. Figure 1 and Figure 2 show the improvement of apparent modulus in compression test and fracture energy in tensile tests, respectively. It can be noticed that the compression modulus proportionally increases by adding 1% wt, 5% wt and 10% wt of ND-ODA.

The improvement of compression modulus of ND-ODA/PLLA can be explained by a well interconnected ND-ODA particle network to PLLA matrix, which could distribute load more effectively through direct particle contact in the matrix. Another important observation is that due to the good affinity between ND-ODA and PLLA, the dispersion quality was enhanced, resulting in a large contact area and thus a significant increase in the fracture energy and the strain at failure. Additionally, their results have showed that below 5% wt ND-ODA, the crystallinity is reduced; at 7%–10% wt ND-ODA, the crystallinity of PLLA is higher. This could be due to the opposing effect of the diamond core and the ODA chains in ND-ODA. At low ND-ODA concentrations, the observed effect is predominantly due to the diamond nanoparticles, which prevent interaction of the polymer molecules and thus reduce the crystallinity of the polymer. At higher concentrations of ND-ODA, the effect of the ODA chains, which can align themselves with PLLA molecules and thus induce crystallization of PLLA, becomes more pronounced [8].

In another relevant case of study, the addition of nanodiamond particles importantly increased the mechanical properties of poly (l-lactide-co-e-caprolactone) (poly(LLA-co-CL) scaffold. By incorporating different amounts of nanodiamond particles in the range of 1%–50% wt in polylactide modified n-DP (n-DP-PLA), it was observed that the composite scaffolds with 10% ND particles, had six times higher E-modulus in tensile tests [11]. One factor that contributes to the improvement of mechanical properties is the uniform distribution of nanoparticles, and the mechanical or chemical linkages established in the composite matrix. Nevertheless, it has been reported that nanodiamond particles agglomerates easily if the connectivity between nanodiamond particles and the polymeric matrix is poor, and/or the distribution is not homogeneous [8,11,13,14,15].

### 2.2. Hidroxyapatite

Hydroxyapatite (HA) is one of the main component minerals of bone and teeth and it is the calcium phosphate that is the focus of current research and clinical use [16]. The favorable biocompatibility and excellent mechanical properties make this bioactive ceramic a potential biomaterial for bone regenerative medicine [17,18,19,20,21,22,23,24]. HA shows appropriate osteoconductivity and biocompatibility because of its chemical and structural similarity to the mineral phase of native bone [25]. Nylon 6 (N6) is commonly used as a medical polymer in applications such as medical threads and artificial skins [17]. N6 can be easily electrospun within a wide range of process and material parameters [17,26]. However, N6 is not a good biodegradable polymer and several handicaps have been reported regarding its role in the formation of hard tissue scaffolds [27].

Abdal-hay et al. [28] immersed as-electrospun Nylon N6 nanofiber membranes into a suspension of HA at a concentration of 5 and 10% wt. The HA nanoplates significantly improved the Young’s modulus and tensile strength of the composite samples. The Young’s modulus of the scaffolds was improved from 9.8 MPa to 19.2MPa and 35.7 MPa for 5 and 10 wt % scaffold samples, respectively.

According to FE-SEM images [28], the scaffold mats obtained from the solution containing 5% wt HA, showed changes in fiber morphology and formation of spider web-like structure. Scaffold mats with 10% wt HA, presented surface rugosity, as well as the formation of spider web-like structure. Based on the researcher’s comments, the increased ionization of the polymer N6/HA solution may have initiated the formation of true-nano fibers, forming the spider web-like structure [28], which surely helped in supporting and distributing the load applied during the tensile test.

Furthermore, the initial increment in the tensile modulus is attributed to the high degree of alignment of the filler nanoplates on the N6 nanofibers. Additionally, the improvement of mechanical properties of HA/N6 nanocomposites may be credited to the well dispersed HA nanoplates onto the N6 fiber surfaces. Additionally, the formation of strong interfacial hydrogen bonds between HA and N6 nanofibers causing a good stress transfer from matrix to nanofibers [28]. Another possible reason is the micromechanical interlocking (adhesion mechanism related with consecutive demineralization, resin infiltration, and polymer setting [29]), which is likely to be induced between the HA nanoplates and the fiber surface molecules in the composites. 

In another case of study, Ramier et al. [30] combined the electrospinning technique with electrospraying process in order to fabricate nanocomposite scaffold mats of poly(3-hydroxybutyrate) (PHB)/hydroxyapatite nanoparticle (nHA). Interestingly, the deposition of HA nanoparticles by electrospraying produced a fibrous scaffold with higher porosity. Owing to their electrostatic repulsive forces, the presence of electrosprayed nanoparticles that covered the fiber surfaces may prevent a tight layering of PHB nanofibers formed during the electrospinning process, thus generating a looser fiber organization of the scaffold with higher pore content [30].

The results showed that the incorporation of HA nanoparticles into the fibers (PHB/nHA (blend)) improves the mechanical properties of PHB mats with an increase of 67% in elastic modulus and 51% in tensile strength. Those improvement was attributed (proposed by the research group) to the favorable interactions between the polymer matrix and the homogeneous distribution of the HA (bioceramic) nanoparticles within the fibers as a filler. One probable explanation they mentioned is that the increment of mechanical properties for the PHB/nHA (blend) is related to the diameter of its fibers (with a value of 640 ± 80 nm), which is the smallest in comparison with the other two samples: PHB/nHA (spray) 950 nm ± 70 and PHB 950 nm ± 160. The lower fiber diameter may provide a higher ability to absorb energy before breaking [30]. In the other hand, the mechanical properties of PHB/nHA (spray) were lower than the others, probably because of its porosity (77% vs. 61% PHB/nHA (blend) and 61% PHB). It was suggested that higher porosity leads to weaker interactions between the constitutive fibers and consequently a decrease of mechanical properties [30].

Another relevant case of study is the influence of a hydroxyapatite (HA) and polycaprolactone (PCL) nanocomposite coating for a scaffold made of biphasic calcium phosphate (BCP) [18]. The results turn out to be very important because the shape (needle, spherical and rod) and size (micro and nano) played a significant role on the improvement of the mechanical properties. The compressive strength coated with nanocomposites was much higher (about 10-20-fold) than the uncoated BCP scaffolds. The highest strength was achieved for the scaffolds with needle shape and nano size (2.1 MPa), with a value of twenty times higher than that of the pure HA (0.1 MPa) and 7 times higher than that of microncomposite [18]. SEM images showed the nanocomposite coating was well bonded to the surface of the scaffold even at the breaking point, suggesting that the interfacial bonding plays an important role in strengthening and toughening the ceramic-polymer composites [18].

### 2.3. Bioactive Glass Particles

Bioactive glass is a subset of inorganic bioactive materials. Due to its excellent biocompatibility and bioactivity it has been studied extensively [19,31,32,33,34]. One example is the study of Esfahani et al. [33]. They coated the struts of a Biphasic calcium phosphates (BCP) scaffold with a nanocomposite layer consisting of bioactive glass nanoparticles (nBG) and polycaprolactone (PCL) (BCP/PCL–nBG) to enhance its mechanical and biological behavior. The effect of nBG concentrations (1–90 wt %) on the mechanical properties and in vitro behavior of the scaffolds was comprehensively examined and compared with a BCP scaffold coated with PCL and hydroxyapatite nanoparticles (nHA) (BCP/PCL–nHA) and a BCP scaffold coated with a single PCL layer (BCP/PCL).

The results demonstrated that the BCP scaffolds were able to withstand a maximum compressive stress of 0.1 MPa, while those coated with a nanocomposite layer showed a compressive strength in the range of 0.2–1.45 MPa, (from two to fourteen times stronger) and the range of 0.24–2.1 MPa (from two to twenty one times stronger) with the addition of nBG and the incorporation of nHA.

BCP/PCL scaffolds showed a compressive strength of 0.3 MPa approximately. Incorporation of nHA and nBG showed a significant increase in the elastic modulus compared with BCP and BCP/PCL scaffolds. The highest compressive strength (increased approx. 14 times) and modulus (increased approx. 3 times) were achieved when 30 wt % nBG was added compared with BCP scaffolds. In summary, the introduction of 1–90 wt % nBG resulted in scaffolds with compressive strengths in the range of 0.2–1.45 MPa and moduli in the range of 19.3–49.4 MPa [33].

After the observation of the substrate surface, they realized that there was no sign of detachment of the nanocomposite layer from the substrate surface, which indicates a strong interfacial bonding of the coating layer to the substrate. The nanocomposite layer stretched considerably before breaking off and the coating presented a ductile fracture surface due to the excellent ductility of PCL [33].

### 2.4. Nano SiO_2_ and MgO Particles

Nano SiO_2_ and MgO particles have special characteristics for bone regenerative applications, they can promote important cellular functions that are required in the implantation such as, proliferation, differentiation as well as mineralization [35,36]. Gao et al. [37] incorporated nanoparticles of SiO_2_ and MgO into β-tricalcium phosphate (β-TCP) scaffolds to increase the mechanical properties (and biological features which are not discussed in this paper). Porous cylindrical β-TCP scaffolds doped with 0.5 wt % SiO_2_, 1.0 wt % MgO, 0.5 wt % SiO_2_ + 1.0 wt % MgO were fabricated via selective laser sintering [37].

According to their results, the concentration of 1.0 wt % MgO increased the compressive strength of β-TCP scaffold more than the concentration of 0.5 wt % SiO_2_. Nevertheless, the compressive strength of β-TCP scaffold doped with SiO_2_/MgO showed the maximum strength increase from 3.12 ± 0.36 to 10.43 ± 0.28 MPa. Higher compressive strength for β-TCP/MgO and β-TCP/SiO_2_/MgO scaffolds might be attributed to the reduced formation of α-TCP phase and increased density after MgO doping [37].

### 2.5. Silver Nanoparticles

Silver nanoparticles (AgNPs) are becoming more and more popular in recent years for biomedical applications due to their antimicrobial activity as well as its good biocompatibility and anti-bacterial affinity [38,39,40]. Mandal et al. [41] coated silver nanoparticles with poly(ethylene) glycol (PEG) and TritonX-100 (TX), and then used the coated nanoparticles in Collagen scaffolds. 

TX is a non-ionic surfactant that has a hydrophilic polyethylene oxide chain and it has been frequently used to extract the lipid like materials from the biological cell membranes [41]. Using different concentrations (0.3, 0.6 and 0.9 mM) PEG and TX, mixed PEG/TX systems with equimolar concentrations capped silver nanoparticles were obtained. It was found that the best combination of PEG and TX was found in 0.9 mM PEG + 0.9 mM TX with a maximum elongation percentage of 46.67%. Nevertheless the highest tensile strength of 0.5 MPa was achieved in 0.6 mM PEG + AgNPs + Collagen Scaffold (CS). SEM images showed how the AgNPs’ morphology changes at different concentrations of PEG/TX coating, which is related to non-agglomerated particles into the Collagen scaffold.

In summary, the incorporation of nanoparticles, coatings, or both to biopolymers to fabricate composite scaffold can significantly enhance the mechanical properties of the scaffolds for bone tissue engineering applications. Table 1 summarizes the results presented in this review; it could provide a guideline for further research and practical applications.

## 3. Conclusions and Recommendations

Mechanical properties of a scaffold are of importance to bone tissue engineering. In the review, current developments in employing nanoparticles and/or coatings to improve the mechanical properties of scaffolds have been examined and reviewed, showing the encouraging progress in this area. In this section, we highlight the most important findings drawn from this review.

1. Good affinity between nanoparticles and scaffold is the key to enhance the tensile strength. Additionally, the stronger interfacial bonding of the coating layer to the substrate can result in higher compressive strength and load transfer efficiency.
2. Good dispersion of nanoparticles can result in a large interfacial area and thus significantly increases fracture energy and other mechanical properties.
3. A thicker coating usually results in a mechanically stronger scaffold.
4. Tensile testing requires gripping the scaffold; bioreactor grips could damage the sample, generating cracks before the measurement. It is a major issue for characterizing the mechanical property of porous ceramic scaffolds using conventional methods.

Based on the review, we would recommend the following future studies.

1. The concentration-dependent effects of nanoparticles on the initiation and propagation of cracks due to scaffolds crystallinity need further, yet systematic, investigation.
2. The influence of size and shape of nanoparticles either as a particles or embedded into coatings, on the mechanical properties of the scaffold is urged to be studied.
3. It would be interesting to look into the relationship between the fiber diameter and the mechanical properties of fibrous scaffolds.
4. It would be essential to investigate into how the chemical affinity between nanoparticles and scaffold materials affects the scaffold mechanical properties.
5. Regarding to mechanical testing, it is necessary to observe in depth the propagation of cracks during compressive or tensile tests and to consider the distribution of nanoparticles (take into account possible agglomeration zones and zones with less concentration of nanoparticles), or other factors that might contribute to the mechanical performance of the scaffolds.
6. Toughness measurement is essential since toughness is a key mechanical property.
7. It could be beneficial to have a comprehensive understanding of how the viscosity and adhesion of the coating affect the coverage and thickness of the coatings and resulted mechanical properties of the scaffolds.
8. Finally, it is important to study the mechanical properties under simulated in vivo environments. Testing mechanical properties in such an environment will allow one to gain insight into the mechanical performance of scaffolds once implanted into patients.

## Figures and Tables

**Table 1:** Summary of mechanical properties studies by using nanoparticles and coatings for bone application.

Reference	Nanoparticle/Coating	Scaffold	Ratio	Test	Results
Zhang et al. (2012) [8].	Nanoparticle octadecylamine-functionalized nanodiamond (ND-ODA)	poly(l-lactic acid) (PLLA)	10% wt ND-ODA/PLLA	Compression MTS servo-controlled hydraulic system, (MTS Systems Co., Eden Prairie, MN, USA) strain rate of 1 mm/min. Tension Instron Testing system, (Instron Co., Norwood, MA, USA) strain rate of 1 mm/min	Strain increase 280% at failure and a 310% increase in fracture energy in tensile tests.
Sun et al. (2015) [11].	Nanodiamond (n-DP)	(l-lactide-co-e-caprolactone) (poly(LLA-co-CL))	10 wt %. n-DP-PLA	Tension Instron 5566 instrument (Instron, UK) with the crosshead speed of 100 mm/min	Increasing E-modulus by about six times (313.6 MPa).
Abdal-hay et al. (2014) [17].	Coating Hydroxyapatite (HA) nanoplates	Nylon N6 nanofibers	N6 nanofibers immersed in a suspension solution of HA powder of 0.5% wt	Tension Tabletop tensile tester (Instron LLOYD Instruments, LR5K Plus, UK) speed 10 mm/min	The Young’s modulus of scaffold was improved by about 225% (average) and the tensile strength was also improved by about 71.8% (average) scaffold samples.
Ramier et al. (2014) [30].	Nanoparticle Hydroxyapatite nanoparticle (nHA)	Poly(3-hydroxybutyrate) (PHB)	14% (wt/vol) nHA/PHB	Tension Instron 5965 (Instron, Norwood, MA, USA) speed of 1mm/min	The mechanical properties of PHB mats with an increase of 67% of the elastic modulus and 51% of the tensile strength at break.
Esfahani et al. (2011) [33].	Bioactive glass nanoparticles (nBG) Composition: 58 mol % SiO_2_, 38 mol % CaO and 4 mol % P_2_O_5_	Biphasic calcium phosphate (BCP) scaffold	30 wt % of nBG in BCP scaffold	Compression Universal Testing Machine (Instron 8874, UK) with a ramp rate of 0.5 mm/min.	The maximum compressive strength (increased aprox. 14 times) and modulus (increased aprox. 3 times) were achieved when 30 wt % nBG was added, compared with BCP scaffolds.
Esfahani et al. (2010) [18].	Composite coating of Hydroxyapatite (HA) and polycaprolactone (PCL)	Biphasic calcium phosphate (BCP) scaffold	3/10% wt. HA/PCL, Nano HA(Needle shape)	Compression Universal Testing Machine (Endura TEC, ELE 3400, Bose,, Eden Prairie, MN, USA) ramp rate of 0.5 mm/min.	The highest strength value was 2.1 MPa with a value 20 times higher than that of pure HA (0.1 MPa).
Gao et al. (2015) [37].	Nano SiO_2_ and MgO particles	β-tricalcium phosphate (β-TCP) scaffolds	0.5 wt % SiO_2_/β-TCP, 1.0 wt % MgO/β-TCP, 0.5 wt % SiO_2_ + 1.0 wt % MgO/β-TCP	Compression Mechanical tester (WD-D1, Shanghai Zhuoji Instruments Co., Shanghai, China) with a constant cross-head speed of 0.4 mm/min.	Improvement from 3.12 ± 0.36 MPa (β-TCP) to 5.74 ± 0.62 MPa (β-TCP/SiO_2_), 9.02 ± 0.55 MPa (β-TCP/MgO), and 10.43 ± 0.28 MPa (β-TCP/SiO_2_/MgO).
Al-Munajjed et al. (2008) [42].	Calcium-phosphate coating	Collagen	0.5 M concentration of the coating, 22 h immersing time	Compression Uniaxial testing system (Zwick Z005 with a 5 N load cell) in phosphate buffered saline (PBS)	Increasing from 0.3 kPa (pure collagen scaffold) to up to 90 kPa (coated scaffold).
Koshkaki et al. (2013) [26].	Beta tricalcium phosphate (b-TCP)	Gelatin	From 10 and 20 wt % of b-TCP nanoparticles	Compression Testing machine (DTM, Zwick-roell, HCT 400/25, Ulm, Germany) at a constant rate of 1 mm min-1 in dry condition.	The Gelatine scaffold had a compressive modulus of 265.8 ± 14. By adding 10 and 20 wt % nano b-TCP, the modulus values increased to 272.6 ± 48 and 429.1 ± 62.2 MPa.
Foroughi et al. [21].	poly-3-hydroxybutyrate (P3HB)	50% wt Hydroxyapatite (HAp)	0.6 g P3HB g/10 mL chloroform, HAp scaffolds were immersed in the polymer solution for 30 s.	Compression tester (SANTAM-Eng. Design Co. Ltd.). The crosshead speed was set at 0.5 mm/min.	The compressive strength without polymer coating was 0.11 MPa, while the compressive strength level of HAp scaffolds with polymer coating was 1.46 MPa.
Esfahani et al. [19]	Bioactive powder, composition: 58 mol % SiO_2_, 38 mol % CaO and 4 mol % P_2_O_5_	Hydroxyapatite (HA)	Bioactive glass coating on HA and sintering at 1000 °C for 2 h.	Compression universal testing machine (AG-400NL, Shimadzu Co.,Kyoto, Japan) at a crosshead speed of 0.5 mm/min.	From 0.22 to 1.49 MPa.
Esfahani et al. [20]	Nanofibrous structured silk over a thin poly(e-caprolactone) (PCL) layer	40% wt Hydroxyapatite(HA)/60% wt Biphasic calcium phosphate (BCP) scaffold	7 wt % silk/HA/β-TCP	Compression Universal testing machine (Instron 8874, UK) with a ramp rate of 0.5 mm/min	The compressive strength and modulus of the modified scaffolds reached 0.42 MPa (compared with 0.07 MPa for BCP) and ≈25 MPa (compared with 5 MPa for BCP), respectively.
Mandal et al. [41]	Coating of poly(ethylene) glycol (PEG) and TritonX-100 (TX) over nanoparticles of silver	Collagen	0.9 mM PEG + 0.9 mM TX	Tension testing machine (SATRA Co., UK, Model No. TM-43 at 20 °C with 65% relative humidity).	Maximum percentage elongation of 46.67%. Application: Implants, catheters and wound dressing materials.

## Abbreviations

The following abbreviations are used in this manuscript:

NDs	Nanodiamonds
PLLA	Poly(l-lactic acid)
ND-ODA	Octadecylamine-functionalized nanodiamond
HA	Hydroxyapatite
N6	Nylon 6
PHB	Poly(3-hydroxybutyrate)
nHA	Hydroxyapatite nanoparticle
PCL	Polycaprolactone
BCP	Biphasic calcium phosphate
nBG	Bioactive glass nanoparticles
β-TCP	β-tricalcium phosphate
AgNPs	Silver nanoparticles
TX	TritonX-100
PEG	Poly(ethylene) glycol
CS	Collagen scaffold
